# A Microfluidic Chip with Double-Slit Arrays for Enhanced Capture of Single Cells

**DOI:** 10.3390/mi9040157

**Published:** 2018-03-31

**Authors:** Jingyi Xu, Shulei Chen, Dongyang Wang, Yue Jiang, Ming Hao, Guangyu Du, Dechun Ba, Qiao Lin, Qi Mei, Yingchao Ning, Da Su, Kun Liu

**Affiliations:** 1 School of Mechanical Engineering and Automation, Northeastern University, Shenyang 110819, China; 1700461@stu.neu.edu.cn (J.X.); 1710120@stu.neu.edu.cn (S.C.); wdysend@gmail.com (D.W.); 1600460@stu.neu.edu.cn (Y.J.); 1500445@stu.neu.edu.cn (M.H.); gydu@mail.neu.edu.cn (G.D.); dchba@mail.neu.edu.cn (D.B.); 2 Department of Mechanical Engineering, Columbia University, New York, NY 10027, USA; qlin@columbia.edu; 3 Department of Oncology, Tongji Hospital, Tongji Medical College, Huazhong University of Science and Technology, Wuhan 430030, China; 4 Shenyang Aeroengine Research Institute, Aviation Industry Corporation of China, Shenyang 110015, China; 1700459@stu.neu.edu.cn; 5 China National Heavy Duty Truck Group Co., Ltd., Jinan 250101, China; suda@jszx.com

**Keywords:** microfluidic chip, hydrodynamic cell trapping, single cell, cell capture, drug delivery

## Abstract

The application of microfluidic technology to manipulate cells or biological particles is becoming one of the rapidly growing areas, and various microarray trapping devices have recently been designed for high throughput single-cell analysis and manipulation. In this paper, we design a double-slit microfluidic chip for hydrodynamic cell trapping at the single-cell level, which maintains a high capture ability. The geometric effects on flow behaviour are investigated in detail for optimizing chip architecture, including the flow velocity, the fluid pressure, and the equivalent stress of cells. Based on the geometrical parameters optimized, the double-slit chip enhances the capture of HeLa cells and the drug experiment verifies the feasibility of the drug delivery.

## 1. Introduction

Microfluidic technology has become a research hotspot in recent years [1], and has received significant attention in various fields, including biomedical science [2] and biochemical studies [3]. Among these fields, cell biology is an important field of microfluidic technology application [4]. Due to the heterogeneity of cells [5], it makes single cell analysis significantly meaningful to study the differences.

In the field of single cell analysis, it is necessary to obtain a large number of single cells for statistical analysis. Compared with conventional methods [6], microfluidics can meet this request of high-throughput single cell analysis. Furthermore, it has various advantages, such as reagent volume control, cell handling, device automation, and multiple component integration [7]. Therefore, microfluidic chip technology in single cell analysis is widely used for intercellular dynamics research, drug screening, and immune response, which greatly promotes the progress of biomedical and cellular biology.

The first critical step for single cell analysis is single cell capture. Currently, a variety of approaches have been reported to capture single cells on microfluidic chips, including dielectrophoresis (DEP) [8], patch clamping [9], optical tweezer [10], and hydrodynamic trapping [11]. Among these methods, hydrodynamic trapping has shown various advantages, including easy operation, high biocompatibility, and high trapping efficiency (passively) [12].

In hydrodynamic trapping, only the fluid force appears during the operation. Cells in the fluid are trapped by similar-size microstructures in the microfluidic device by hydrodynamic effects. To effectively capture cells, various cell trap structures are proposed. A bowl-shaped micro-trap structure was designed for single cell capture and this structure was used to capture individual cells that were used to study cell attachment, death, and division [13]. The microfluidic device based on the hydrodynamic trapping of cancer cells in controlled geometries was developed, and the formation of spheroids was enhanced by maintaining compact groups of the trapped cells due to continuous perfusion [14]. Subsequently, the researchers presented a microfluidic device containing a dense array of weir-based passive hydrodynamic cell traps. Using a novel geometry and a three-step loading protocol, it could immobilize and pair thousands of cells immediately [15]. Furthermore, the researchers developed a microfluidic capture system for single cell detection that had the ability to monitor cell dynamics over time [16].

However, in previous studies, few researchers have focused on the geometric effects on the flow field in microfluidic chips. Since some important controllable variables have not been systematically studied, the capture efficiency of many single cell capture experiments in microfluidic chips is unstable, and is not conducive to subsequent manipulation such as cell culture, cell analysis, and cell dose. Therefore, it is critical to study these controllable variables. Investigations of flow behaviour in the micro-environment are not only beneficial to a better understanding of capture process, but also makes the process more controllable. To optimize the efficiency, a number of simulation works have been carried out. Computational fluid dynamics (CFD) can simulate complex and coupled physics rapidly at low cost [17,18,19,20,21,22]. Researchers use CFD simulation to investigate hydrodynamic trapping mechanisms and cell mechanical environments [22]. 

In this paper, we designed a double-slit microfluidic device for hydrodynamic cell trapping at the single cell level, and the mechanism of hydrodynamic trapping was applied to study flow behaviour in the microfluidic chips. To optimize the chip design and implement basic research, the geometric effects on flow behaviour in the chip micro-environment were analysed in detail. Then, the double-slit chip was geometrically optimized for enhanced capture of HeLa cells and the feasibility of the drug delivery function was verified. The study established a method to explore more practical microfluidic chip architectures, and provided reference and guidance to further study in cell-trap flow behaviour, chip optimization, and structure innovation.

## 2. Problem Formation

**Figure 1:**
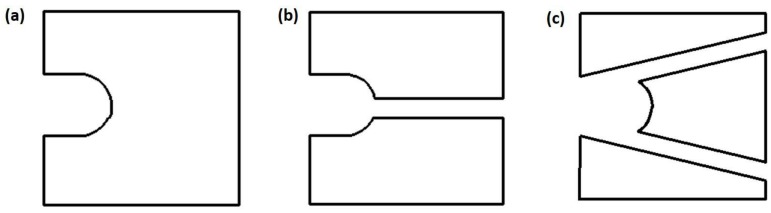
The schematic diagram of three types of capture structures: (**a**) seamless; (**b**) single-slit; (**c**) double-slit.

The main purpose of the cell capture chip is to enable the capture and retention of cells. Therefore, it is critical to design the capture structure with good effect, and its structure should match the trapped particle geometry. A series of index parameters are also required to be taken into consideration, including the capture efficiency and loading time of the cell, the pressure and velocity distribution of the fluid, the deformation, and the equivalent stress of the cell. Based on the hydrodynamic cell capture method [23] and our previous study [24,25], three types of microstructures were designed, including seamless, single-slit, and double-slit traps, as shown in Figure 1.

In a previous study, the researchers designed a microfluidic chip for the capture and signal transmission analysis of hematopoietic stem cells, enabling parallel analysis of hundreds of single cells [26]. This demonstrated that double-slit arrays enhanced the capture of living hematopoietic cells. Furthermore, in our previous studies [27], it was found that the double-slit trap structure had better capture ability compared to seamless and single-slit structures. In the seamless chip, the cells could enter into the micro-trap at a high inlet velocity, making the internal pressure of the chip larger, easily causing leakage and other issues. In the single-slit chip, the critical velocity was greatly reduced compared to the seamless chip. However, due to the presence of the single slit, the cell was accelerated and the equivalent stress increased sharply as well, resulting in greater deformation and damage.

Therefore, this study focuses on the double-slit chip design. The geometry of a single trap and its surrounding microfluidic channels were optimized using finite element method (FEM). Compared with the work by Wlodkowic et al. [26], the angle of the double slit designed by us had a certain inclination to make the fluid flow stable, which was also beneficial to enhance the possibilities of capture. Meanwhile, we focused on single cell capture and the optimization of micro-traps. The behaviour and mechanism of flow were investigated to enhance capture ability. Due to the inconsistent size of the individual cells, the cells were considered to be microspheres with a radius of 5 μm in the process of calculation and simulation. Because of the high computational demand in 3D fluidic dynamics simulations, the simulations were done in 2D. Polydimethylsiloxane (PDMS) walls were considered to be rigid, and the fluid was considered to be incompressible when simulating fluid—structure interaction.

## 3. Materials and Methods

### 3.1. Design and Fabrication of the Microfluidic Chip

**Figure 2:**
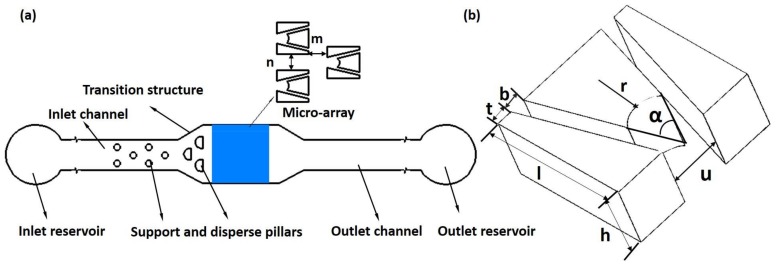
The schematic diagram of the chip: (**a**) the overall structure; (**b**) the double-slit structure.

All parts of microfluidic device were designed by Auto-CAD and fabricated using the standard soft lithography method [28]. The schematic diagram of the overall structure of the chip is shown in Figure 2a. The structure of the chip was designed into a narrow shape, including inlet and outlet reservoir, support and disperse pillars, channel, microarray, and transition structure. The inlet and outlet reservoirs are used for fluid transition, and avoid fluid flowing directly into the chip. Because their areas are larger than inlet and outlet, it ensures the stability of fluid flow within the chip. In the inlet channel, the cells are separated by the dispersed strands so that they can enter into the capture array more uniformly. The design of the transition structure is mainly for the connection of the inlet and outlet channels with the capture area, which can avoid the generation of vortices owing to the different cross-sectional area of their channels and the capture area.

In the trap array device, the number of micro-traps was designed to be 351 distributed in 26 rows, while the number of micro-traps in each row was 13 in odd rows or 14 in even rows. Each row of traps was offset horizontally with respect to the one above it. This offset ensures that the trapping function of the next row is not influenced by the row above it. The double-slit microarray geometry is shown in detail in Figure 2b.The radius of the microsphere is defined as *R*; the radius of the groove as *r*; the height of the microarray as *h*; the length and the bottom width of the groove walls as *l* and *t*, respectively; the opening angle of the trap as α; the widths of the trap opening and double-slit as *u* and *b*, respectively; and the horizontal and vertical spacing of trap arrays as *m* and *n*, respectively. The spacing ratio was defined as *m*:*n*.

### 3.2. Computational Models and Trapping Mechanism

Computational fluid dynamics (CFD) was applied to investigate the flow field in the micro-environment. For modelling the double-slit chip, the incompressible Navier–Stokes equations with a continuity equation were employed to determine the fluid flow as follows:

(1) $$\frac{\partial u}{\partial x}+\frac{\partial v}{\partial y}=0,$$

(2) $$u\frac{\partial u}{\partial x}+v\frac{\partial v}{\partial y}=-\frac{1}{\rho }\frac{\partial p}{\partial x}+\mu (\frac{\partial^{2}u}{\partial x^{2}}+\frac{\partial^{2}u}{\partial y^{2}}),$$

where *ρ*, *p*, and *μ* are the density, pressure, and kinematic viscosity, respectively. *u* and *v* are the velocity components in *x*- and *y*-directions.

**Figure 3:**
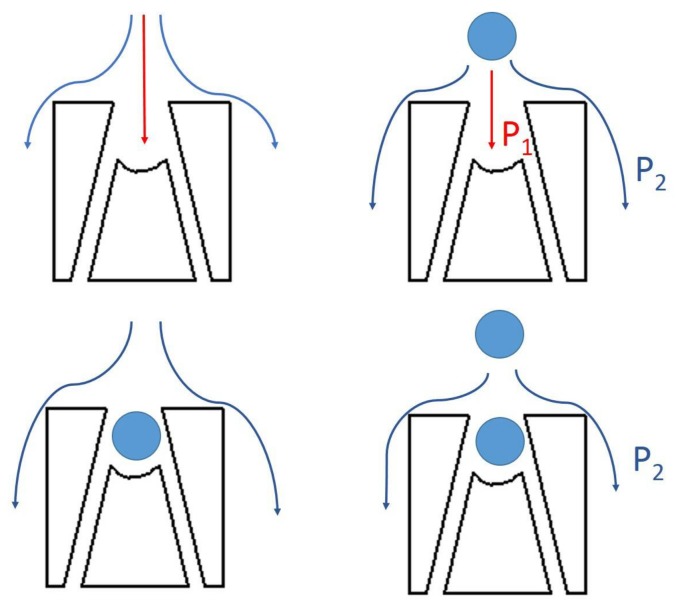
Schematic diagram of the cell capture process based on the hydrodynamic method.

The chip uses the hydrodynamic trapping method to capture cells and employs fluidic resistance engineering to achieve it [29,30,31]. Therefore, it is critical to study the hydrodynamics trapping mechanism. The schematic diagram of cell capture process based on the hydrodynamic method is shown in Figure 3. Path P_1_ (red line) and path P_2_ (blue line) are the trapping and bypassing paths, respectively. Its main capture process is mainly divided into four processes: fluid flow, cells before entering, cells entering, and bypassing. Before filling the cell suspension, the flow of the pure fluid is dominant in the chip and some of the fluid enters into the micro-trap. Because the cells generally follow the streamline within the channel, once the cell enters into the micro-trap, the corresponding streamline is cut off and the next cell becomes difficult to access, bypassing the trap under the action of the fluid. Therefore, the array geometry should be designed so that the trapping path P_1_ for an empty trap has a lower flow resistance than the bypassing path P_2_.

Based on the optimization method proposed by Xu et al. [20], the microfluidic cell-trap arrays should ensure a single cell in each trap and satisfy other design criteria, such as avoiding channel clogging and multiple microspheres trapping at one trap location, satisfying the trap array device’s micro-fabrication tolerance and feasibility, and obtaining stable trapping of the cells.

Therefore, the geometry of the double slit should be limited within an appropriate range. The value of *b* was required to be smaller than the microsphere’s diameter, and *u* should be smaller than the sum of two microsphere diameters, which ensured that a microsphere would be trapped in a trap and reduced the chance that multiple microspheres would be in one trap. The value of *h* should also be larger than one microsphere’s diameter to guarantee the microsphere would be immobilized in the trap. The value of *r* was required to be larger than the radius of the microsphere to ensure that a microsphere would be retained in a trap and not swept away due to the transient flow motion around the trap. The value of *n* should be larger than the diameter of one microsphere to allow others to flow through the channel during the bypassing process. For fabrication feasibility, the values of *t* and *l* should be limited within an appropriate range. Too small a value of *t* is difficult to fabricate using soft lithography and too large a value of *l*/*t* leads to easy collapse.

Therefore, the geometric parameters for the microfluidic microsphere-trap array were fixed as in Table 1.

### 3.3. Materials and Cell Preparation

Dulbecco’s Modified Eagle Medium (DMEM), foetal bovine serum, penicillin-streptomycin, trypsin- trypsin-Ethylenediaminetetraacetic acid (EDTA), and HeLa cells were all purchased from Sangon Biotech Co., Ltd., (Shanghai, China). Polystyrene microspheres were purchased from Tianjin Beisi Technology Development Center (Tianjin, China). Cisplatin, phosphate buffered saline (PBS), and trypan blue dye were obtained from Tongji Hospital (Wuhan, China). Cisplatin powder (10 mg) was mixed with 100 mL of 0.9% normal saline and dissolved. Trypan blue of 0.4% stock solution was diluted with PBS to a final concentration of 0.04% in 9:1 ratio.

HeLa cells were cultured in DMEM with 10% foetal bovine serum and 1% penicillin-streptomycin. Cells were cultured in a 37 °C incubator under 5% CO_2_ and passaged every 2 days by dissociation with 0.25% trypsin-EDTA. Before use, adherent cells were harvested by trypsinization with 0.25% trypsin-EDTA at 37 °C and centrifuged at 800–1000 rpm for 3 min. The cells were then suspended in fresh supplemented DMEM for use.

### 3.4. Microsphere and Cell Manipulation

The microfluidic platform was built to utilize microfluidic chips for microsphere and cell manipulation. The cell capture experiment platform was detailed as demonstrated in Appendix A.

Two microchips were needed in this experiment. The microchips were first sterilized with 75% ethanol for 5 min prior to loading. Sterile PBS was then used to fill the device. Mixed with 0.8 mL of polystyrene suspension and 4 mL of deionized water, the suspension was introduced into the chip at a rate of 0.1 μL/s. The cell suspension was introduced at a rate of 0.1 μL/s into the other chip for 5 min to achieve a stable capture effect. After cells were trapped to the desired density, cisplatin diluted with culture medium was introduced into this chip. After the drug was completely absorbed, it was added to the prepared trypan blue dye and stained for about 3 min. Microspheres and cells in the microfluidic chip were imaged on a display through a microscope.

## 4. Results and Discussion

Considering the slight effect of other sizes on the double-slit structure and machining accuracy, the optimization was focused on the values of spacing and opening angle. According to the results of optimization, the fluid distribution in the chip was studied in detail.

### 4.1. The Optimization of Micro-Trap Spacing

**Figure 4:**
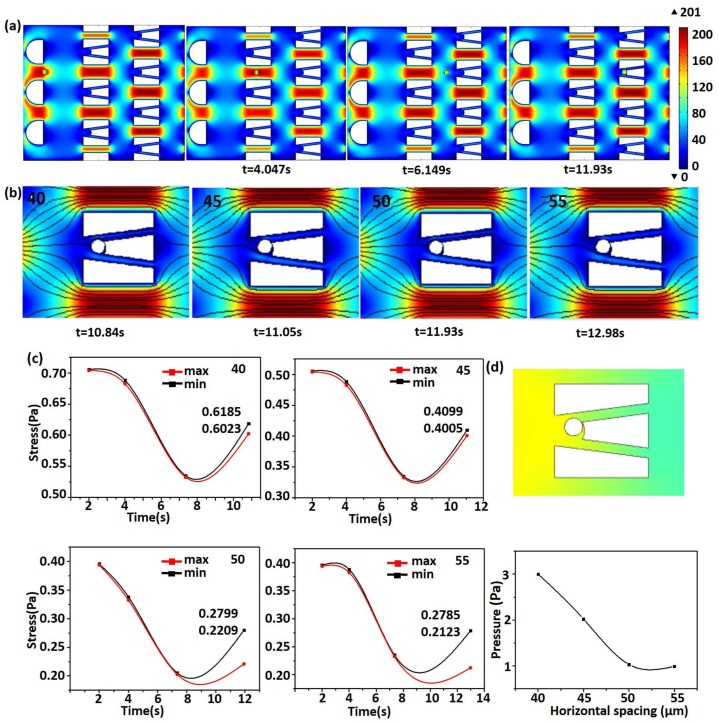
(**a**) The movement of the microsphere at the horizontal spacing of 50 μm; (**b**) The final capture of the microsphere in different horizontal spacings (40, 45, 50, 55 µm, left to right); (**c**) The stress of the microsphere in different horizontal spacings; (**d**) The fluid pressure in different horizontal spacings.

First, the parameter *h* was set to be 15 μm, for microspheres of radius 5 μm. Based on experimental testing results, we chose *h* = 3*r* and the spacing ratio *m*:*n* = 2:1. As shown in Figure 4, the horizontal spacing and opening angle were fixed as 40 μm and 25° at first, and its horizontal spacing ranged 40 μm from 55 μm. The inlet velocity was selected as 10 μm/s at first.

Figure 4a shows the movement of the microsphere in the chip at the horizontal spacing of 50 μm. The microsphere moved close to the micro-trap at 6.149 s. After a period of movement, the microsphere entered into the micro-trap at 11.93 s. Because fluid flows quickly in contraction areas and slowly in extending areas [32], the flow moved quickly through the gaps among the micro-traps and slowly through the micro-traps. Further, only the flow in close proximity to the microsphere was affected by the microsphere’s motion, and the other areas were not influenced. Therefore, the displacement of the microsphere was mainly along the *x*-axis, while the displacement in the *y-*direction was small.

Figure 4b shows the final capture of the microsphere in different horizontal spacings. The capture time became longer due to the increase of horizontal spacing. With the increase of the displacement of the microsphere’s motion, the loading time of the microsphere became longer. The streamlines also show that there were no vortices in the creeping flow, which ensured the stable flow of fluid and the state of the microsphere. 

However, with the increase of horizontal spacing, the equivalent stress of the microsphere became smaller. The change in the equivalent stress of the microsphere over time is shown in Figure 4c. The equivalent stress can be used to determine whether the microsphere can yield to a complex force. Due to the different value of the force inside the microsphere, the maximum and minimum values are also shown in the figure. When the horizontal spacing increased, the fluid in the chip flowed fully and stably. In the course of the movement, the equivalent stress of the microsphere as well as the fluctuation were relatively small. Only when the microsphere was trapped did the stress on the microsphere increase, because of the force from the micro-trap. When the chip is applied to capture biological cells, the presence of stress can cause cell deformation, fusion, or even death. Therefore, low equivalent stress is critical, which is conducive to maintaining the state of cells.

Finally, the fluid pressure in the chip was studied. Figure 4d shows the fluid pressure in different horizontal spacings. The fluid pressure in the chip decreased with increasing spacing. When the microsphere was close to the micro-trap, the pressure between the microsphere and the micro-trap increased and imposed negative force on the microsphere. When the microsphere was immobilized in the micro-trap, the pressure in the very small gap between the microsphere and the micro-trap became larger than that at the inlet. In order to ensure the stable trapping of the microsphere, stable pressure is critical. The presence of the double slit gives the fluid more choice to flow. When the microsphere was close to the wall of the micro-trap, the compressed fluid could be flowed from the double slit correspondingly. This can relieve the pressure of the fluid imposed upon the microsphere. Furthermore, increasing spacing also contributes to the decrease of the fluid pressure, which is conducive to the state of chips.

### 4.2. The Optimization of the Opening Angle

**Figure 5:**
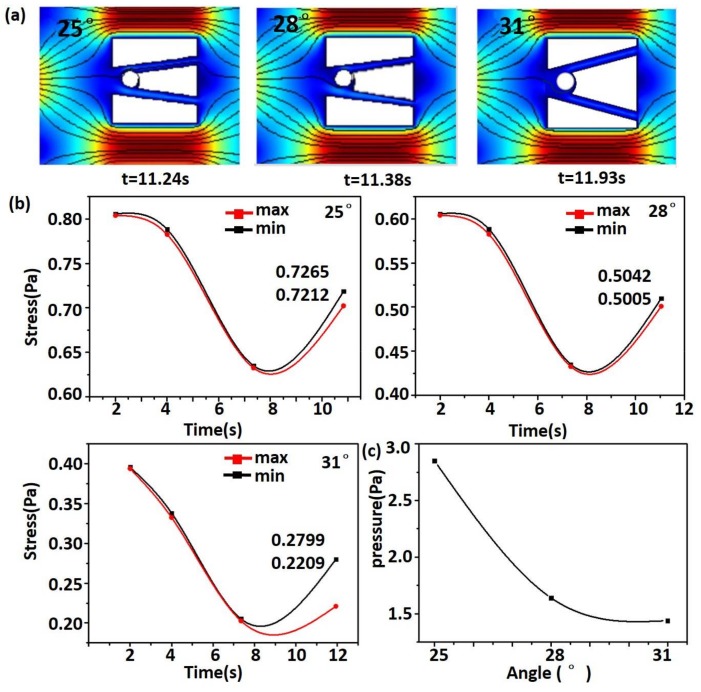
(**a**) The final capture of the microsphere in different angles; (**b**) The stress of the microsphere in different angles; (**c**) The fluid pressure in different angles.

Combined with the above analysis, a microfluidic chip with short capture time and low equivalent stress of the microsphere and fluid pressure was needed. Based on the above discussion, the horizontal spacing was fixed as 50 μm. The optimization of the opening angle was performed as in Figure 5.

As shown in Figure 5a, the opening angle ranged 25° from 31°, and the capture time became longer with the increase of the opening angle. Similarly, as shown in Figure 5b, the equivalent stress of the microsphere was also studied. With the increase of opening angle, the equivalent stress of the microsphere became smaller. The fluid pressure in different angles at the double-slit in the chip is shown in Figure 5c. The fluid pressure decreased with the increase of opening angle.

It can be seen from the figure that the capture time was shortest when *α* was 25°. However, the equivalent stress of the microsphere and the fluid pressure were both high. The presence of sharp corners at the groove resulted in high equivalent stress of the microsphere and the fluid pressure, which is not conducive to the state of cells. When *α* was 28°, the capture time was longer than the angle of 25°. The equivalent stress of the microsphere and the fluid pressure were lower than the angle of 25°, which is conducive to the state of cells and chips. Although the equivalent stress of the microsphere and the fluid pressure were lowest while *α* was 31°, the capture time was longest. Considering its comprehensive effect, *α* was fixed at 28°.

### 4.3. Fluid Velocity Distribution

According to the discussion above, the geometric parameters for the microfluidic microsphere-trap array are summarized in Table 2.

**Figure 6:**
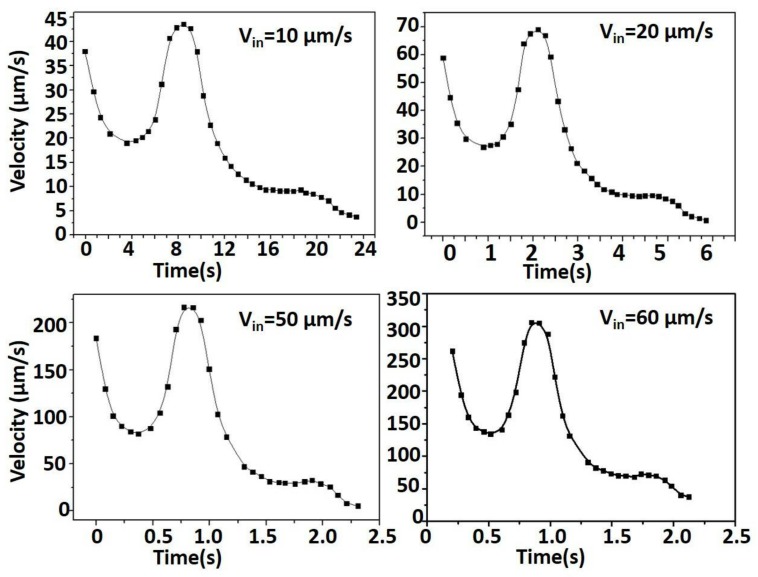
The change of velocity of the microsphere.

For a double-slit chip, the microsphere could be captured at a very low critical rate. The capture time should be fully taken into account to increase the actual efficiency of the chip. Therefore, the inlet velocity was studied. The change of velocity of the microsphere is shown in Figure 6.

At the beginning, the microsphere was located in the middle of the two pillars. With the increase of cross-sectional area, the speed gradually decreased. Then, as the fluid flowed, the speed began to increase. When passing between two micro-traps, the speed of the microsphere reached the maximum value. After the microsphere entered into the micro-trap, it moved at a stable speed, and the final speed dropped to 0, and the microsphere was captured. 

**Figure 7:**
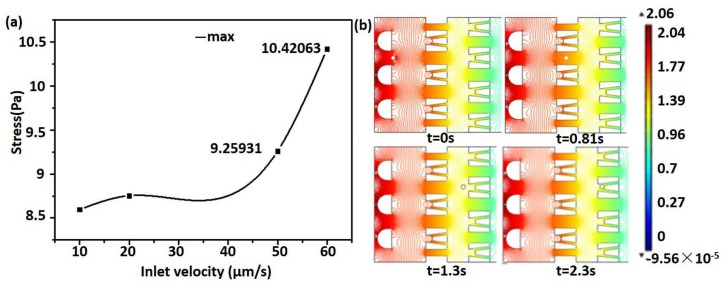
(**a**) The equivalent stress of the microsphere at different inlet velocities; (**b**) The fluid pressure in the chip at a velocity of 50 μm/s.

The microsphere in the chip mainly suffered from fluid resistance and viscous drag force. As the microsphere continuously approached the micro-trap, the resistance increased and the drag force decreased, which made the speed of the microsphere decrease rapidly. When it entered into the interior of the micro-trap, the resistance and the drag remained consistent and the microsphere moved at a stable speed. Then, the resistance was greater than the drag, and the microsphere began to slow down until stopped. With increasing inlet velocity, the speed of the microsphere between two micro-traps became higher and the high speed of the microsphere in the chip was not beneficial to capture cells. It also caused the increase of the equivalent stress of the microsphere and fluid pressure, which made the chip easy to cause leakage. The equivalent stress of the microsphere is shown in Figure 7a.

With the increase of inlet velocity, the equivalent stress of the microsphere also increased. When the inlet velocity was 50 μm/s, the maximum equivalent stress of the microsphere was 9.25931. It was below 10, which is not sufficient to produce deformation of the cells [33,34]. This low equivalent stress is conducive to maintaining the state of cell when capturing them. Furthermore, as shown in Figure 7b, the pressure field of the flow at a series of time points became smaller, which was conducive to the use of chip and cell capture.

### 4.4. Cell-Trap and Drug Delivery in the Microfluidic Chip

**Figure 8:**
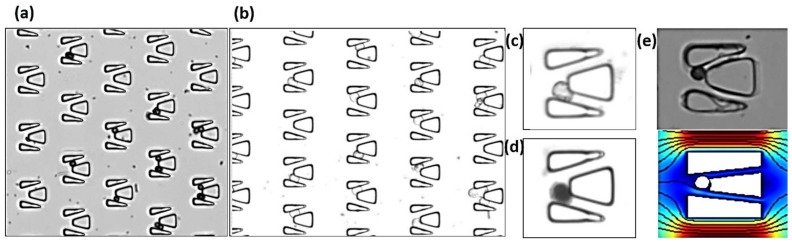
The cell-trap and drug testing results: (**a**) microspheres; (**b**) HeLa cells; (**c**) normal cell; (**d**) drug dosing; (**e**) comparison of experiment and simulation.

The developed microfluidic device was utilized for cell capture and antitumor drug delivery to explore the potential application in clinical oncology. In our experiment, polystyrene microspheres and HeLa cells were introduced into the chips. The size of HeLa cells is basically the same as that of the microspheres. The cell-trap and drug delivery results are shown in Figure 8. The dead cells allowed trypan blue dye to penetrate the cell membrane and disintegrate DNA due to the degeneration of the cell membrane. The entire dead cells were disseminated with a dye solution and turned blue. The living cells and their edges were clear, showing transparency. The stained cells were immersed by blue dye, showing black under the microscope.

As shown in Figure 8a,b, through the capture experiment, it can be found that when the microsphere and the cell entered into the double-slit micro-trap, it moved toward a seam in the double-slit until it stopped. Due to the rigidity of microspheres, they did not deform like cells. As shown in Figure 8c,d, through the drug experiment, it can be found that the living cells and their edges were clear. After drug delivery, the stained cells were immersed by blue dye, showing black under the microscope, effectively illustrating that this chip could capture the cell and verifying the feasibility of the drug delivery function. In addition, it can be seen from Figure 8e that the experimental results were basically the same as the simulation results, reflecting that the double-slit chip could achieve a good capture effect.

However, due to the problem of processing, the design of the micro-trap within the space was larger than the original design, which caused the existence of a few microspheres and cells in the micro-trap, and the microspheres moved into a seam in the double-slit. Otherwise, the deformation of cells also caused the cells move into a seam. During our experiment, it can be found that most micro-traps could capture a single cell. Some micro-traps had two cells, and few micro-traps had more cells. The ratio of cells’ number in micro-traps is shown in Table 3.

## 5. Conclusions

In this study, we designed a double-slit microfluidic chip and investigated a single cell capture process in different microfluidic cell-trap array chips. Through the optimization of spacing and opening angle, the equivalent stress that cells suffered was low and stable. The fluid pressure in the double-slit chip was also low, which was conducive to the state of the cells and the chip. The geometry of double-slits were fixed eventually. According to the results of optimization, we found that the chip had the best capture condition when the inlet velocity was 50 μm/s. 

Furthermore, we conducted an experiment according to the simulation conditions. The experiment illustrated that the double-slit chip could capture single cells and could be used for antitumor drug delivery. Through the drug experiments to verify the feasibility of the drug delivery function of this chip, the establishment of a unit model in microfluidic platform provides research value for the optimization of chip integration and platinum drug development. Future work will continuously concentrate on the optimization of the double slit to achieve better capture effects. Due to the complex flow of fluids in micro-scale and the elastic properties of cells, the real fluidic conditions within the traps were not completely the same as predicted by CFD models. We will also investigate the actual flow behaviour and mechanism in the chip and continuously combine CFD models and experiment.

## Figures and Tables

**Table 1:** The geometric parameters for the microfluidic microsphere-trap array.

**Parameters**	*r*	*h*	*l*	*t*	*b*	*u*	*α*	*m*	*n*
**Values (μm)**	7	15	50	4	5	16	25	40	20

**Table 2:** The geometric parameters for the microfluidic microsphere-trap array.

**Parameters**	*r*	*h*	*l*	*t*	*b*	*u*	*α*	*m*	*n*
**Values (μm)**	7	15	50	4	5	16	28	50	25

**Table 3:** The ratio of cells’ number in micro-traps.

Number	Single	Double	More	Empty
**Ratio (%)**	70	16	8	6

**Figure A1:**
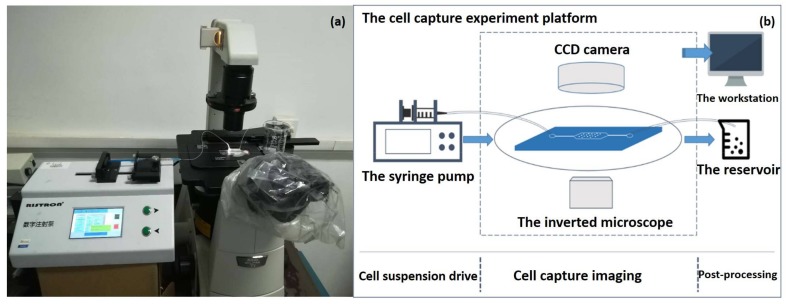
Schematic diagram of the cell capture experiment platform: (**a**) The main equipment used in the experiment; (**b**): The overall cell capture experiment platform.

## Appendix A

A schematic diagram of the cell capture experiment platform is shown in Figure A1. The microfluidic platform built to utilize microfluidic chips for microsphere and cell manipulation consisted of four major parts: (1) An inverted microscope in Particle Image Velocimetry (PIV) System (Beijing Microvec Pte. Ltd., Beijing, China) with a charge coupled device (CCD) camera (Beijing Microvec Pte. Ltd., Beijing, China)was used to observe and photograph the microfluidic channels and microsphere or cell; (2) A computer connected to the CCD camera was used to capture images; (3) A syringe pump (JiaShan Ruichuang Sci-Tec Co., Ltd., JiaShan, China) was used for solution injection; (4) 1 mL disposable syringes and polyethylene (PE) pipes ( Shenyang-Lab Science and Trade Co., Ltd, Shenyang, China) were used for the connection of the device.
